# Exploring the Possible Role of Endometriosis-Associated Dysbiosis in Endometrial Carcinogenesis

**DOI:** 10.3390/medicina62081577

**Published:** 2026-08-17

**Authors:** Costin Vlad Anastasiu, Oana Gabriela Dimienescu, Maria Alexandra Dinuță-Smeu, Marius Alexandru Moga, Ovidiu Dan Grigorescu, Gabriela Gugiu, Alina Bisoc

**Affiliations:** 1 Faculty of Medicine, Transilvania University of Brasov, 500036 Brașov, Romania; 2 Medicine PhD School, Transilvania University of Brasov, 500036 Brașov, Romania

**Keywords:** endometrial cancer, endometriosis-associated dysbiosis, uterine microbiota, gut microbiota, estrobolome, endometrial carcinogenesis, inflammation, tumor microenvironment

## Abstract

*Background and Objectives:* Endometriosis is associated with chronic inflammation, immune dysregulation, oestrogen-dependent growth, oxidative stress, altered steroid hormone metabolism, compromised epithelial barrier integrity, and the production of bioactive microbial metabolites. These interconnected alterations have been proposed to create, in principle, a permissive local microenvironment for malignant transformation. This narrative review examines whether endometriosis-associated dysregulation of the gut and reproductive tract microbiota may act as a hypothetical biological modulator linking these multi-axis changes to endometrial carcinogenesis, with attention to immunological, endocrine, metabolic, microbial–metabolite, oxidative, and barrier-related pathways. *Material and Methods*: We narratively integrated current evidence on gut and reproductive tract microbiota alterations relevant to endometrial homeostasis, with emphasis on the estrobolome, low-biomass uterine microbial communities, inflammatory and immune signaling, microbial metabolites, and pathways implicated in carcinogenesis. *Results:* Available data suggest that dysbiosis may influence endometrial carcinogenesis through interconnected endocrine, inflammatory, metabolic, and immune mechanisms. Attention has been given to loss of Lactobacillus dominance, enrichment of anaerobic and pro-inflammatory taxa, altered estrogen recirculation, progesterone resistance, Toll-like receptor activation, NF-κB/STAT3 signaling, COX-2/PGE2 activity, PI3K/AKT/mTOR pathway activation, oxidative stress, macrophage polarization, and impaired natural killer cell surveillance. These alterations may contribute to a permissive microenvironment characterized by persistent inflammation, defective immune control, and disrupted endometrial homeostasis. However, the current literature remains limited by small and heterogeneous cohorts, predominantly cross-sectional designs, contamination risk, and marked methodological variability, particularly in low-biomass uterine samples. *Conclusions:* Current evidence supports the view that microbiome dysregulation is a context-dependent biological modulator that intersects with endocrine, inflammatory, metabolic, immune, oxidative, and barrier-related pathways relevant to endometrial carcinogenesis. Microbiome dysbiosis should be regarded as a hypothetical contributory factor rather than as an established causal driver. Its near-term translational relevance appears greater for biomarker development and risk stratification than for immediate microbiome-directed therapy. Longitudinal, standardized, and functionally integrated studies are needed to clarify whether microbiome-associated signatures can be translated into clinically meaningful prevention and management strategies in endometrial cancer.

## 1. Introduction

The female reproductive tract is a hormonally responsive mucosal ecosystem in which epithelial barriers, immune surveillance, and microbial communities interact to preserve tissue homeostasis. Under eubiotic conditions, these interactions support barrier integrity, controlled inflammatory tone, and tolerance toward commensal microorganisms, whereas dysbiosis may promote epithelial disruption, aberrant immune activation, and persistent mucosal inflammation. This framework is particularly relevant to endometriosis-associated dysbiosis, in which altered host–microbe crosstalk may contribute to lesion persistence and potentially to a biological context permissive for malignant transformation [1,2,3,4].

Endometrial cancer remains the most frequent gynecologic malignancy in high-income countries, and its incidence continues to rise in parallel with obesity, metabolic dysfunction, and population aging [5]. Although its classical pathophysiologic framework has long emphasized prolonged estrogenic stimulation, progesterone resistance, chronic inflammation, and progressive molecular derangement, contemporary models increasingly recognize that these processes unfold within a broader endocrine-immunometabolism network shaped, at least in part, by the microbiome. In this context, the gut and the female reproductive tract microbiota are increasingly viewed as biologically active regulators of epithelial integrity, steroid hormone turnover, mucosal immunity, and inflammatory tone, all of which are relevant to endometrial homeostasis and carcinogenesis [5,6].

Endometriosis provides a particularly informative biologic model through which these interactions may be interpreted. Although considered a benign gynecologic disease, it shares several features with neoplastic processes, including resistance to apoptosis, invasive behavior, angiogenic activation, oxidative stress, immune evasion, and sustained inflammatory signaling. Epidemiologic evidence most strongly supports an association between endometriosis and ovarian malignancy, whereas the relationship with endometrial cancer appears weaker, more heterogeneous, and likely influenced by histologic subtype, hormonal milieu, and metabolic context. Nevertheless, the coexistence of estrogen dependence, progesterone resistance, immune dysregulation, and persistent tissue injury suggests that endometriosis may create conditions that increase endometrial susceptibility to malignant progression in selected biologic contexts [7,8].

Altered gut and reproductive tract microbial communities may contribute to carcinogenesis through overlapping mechanisms, including epithelial barrier disruption, TLR/NF-κB activation, cytokine amplification, macrophage polarization, altered estrogen recirculation through the estrobolome, and remodeling of the local immune microenvironment [6,9,10,11].

We hypothesized that microbiome dysregulation—when coupled with endometriosis-associated inflammation, altered estrogen metabolism, and impaired immune surveillance—may contribute to the initiation or promotion of endometrial carcinogenesis.

## 2. Materials and Methods

This narrative review evaluates current evidence on the potential role of microbiome dysregulation in linking endometriosis-associated inflammation with endometrial carcinogenesis. A literature research was performed in PubMed and Google Scholar, with emphasis on studies published between 2015 and 2025. Search terms included combinations of “endometriosis”, “microbiota”, “microbiome”, “dysbiosis”, “endometrial cancer”, “endometrial carcinogenesis”, “estrobolome”, “microbial metabolites”, and related pathway-specific terms. Priority was given to original human studies, while experimental and translational reports were included when they clarified relevant mechanistic pathways. Given the heterogeneity of study design, sampling strategies, sequencing approaches, and contamination control in this field, the present work was designed as a narrative review intended to provide a critical synthesis rather than a systematic or quantitative analysis. Whenever possible, greater interpretive weight was assigned to studies with clearer phenotypic characterization, better contamination-aware design, and stronger clinical relevance.

## 3. Endometrial and Uterine Microbiota

For many years, the uterine cavity was regarded as a sterile environment. However, culture-independent molecular studies have progressively challenged this concept by demonstrating that the upper female reproductive tract suggesting the presence of a low-biomass microbial signal or a putative microbial community rather than definitively proving viable resident microorganisms [11,12,13,14,15,16,17]. The ascending migration of bacteria from the vagina through the cervix is considered the most plausible route for endometrial colonization, although it is unlikely to be the only mechanism involved [12,16,17,18]. The discovery of polymicrobial biofilms linked to bacterial vaginosis on the endometrium and fallopian tubes, along with data showing that the cervical mucus plug lessens but does not completely stop the growth of bacteria, lend credence to this theory [17,19]. These findings challenge the traditional concept of an impenetrable cervical–uterine barrier. The endometrium is best viewed as a mucosal niche influenced by local continuity with the lower genital tract and systemic host-microbe interactions [1,12,16].

### 3.1. Composition of the Endometrial and Uterine Microbiota

**Figure 1:**
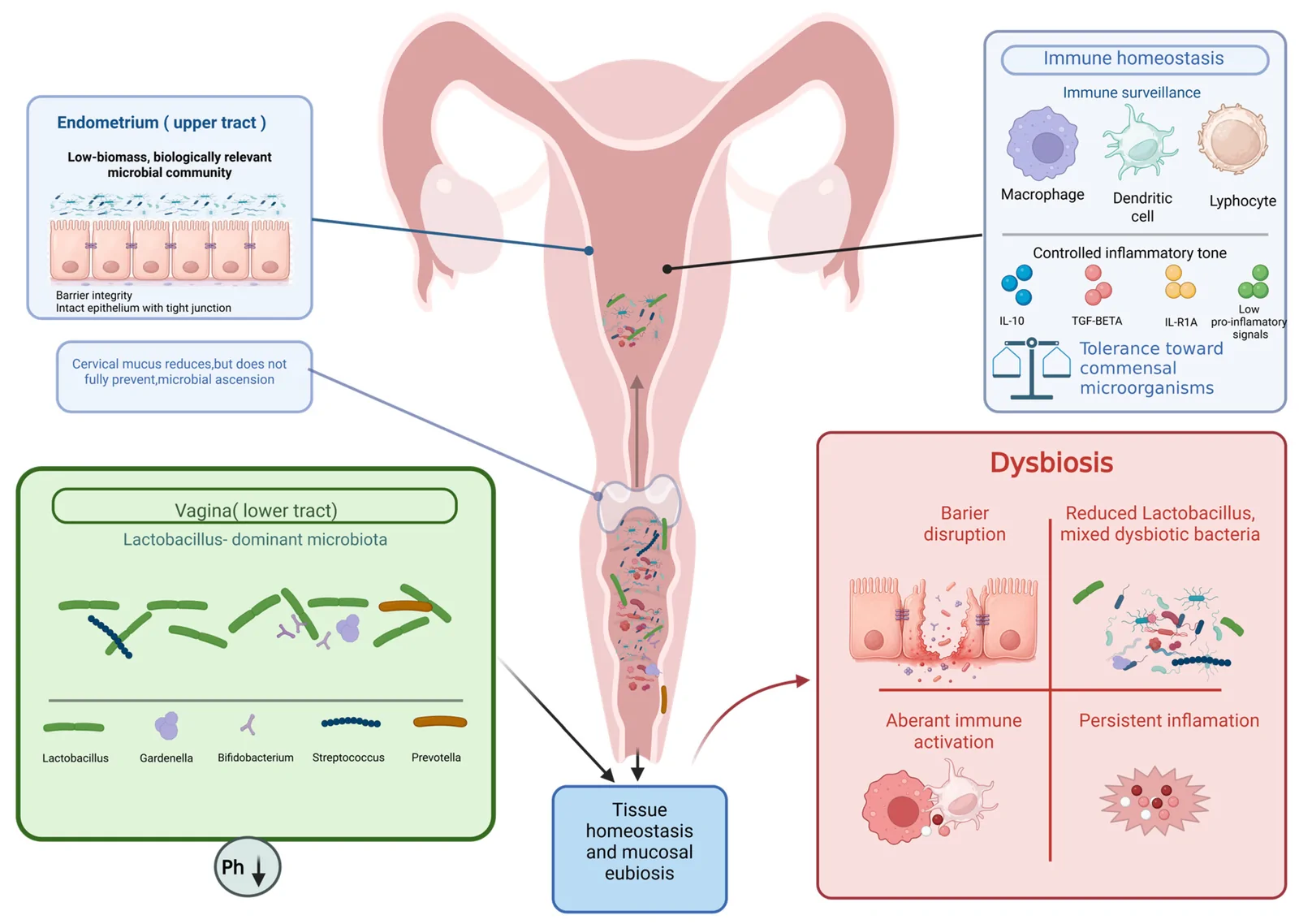
Mucosal homeostasis and microbial eubiosis in the healthy female reproductive tract.

The existence of endometrial microbiota is increasingly established, but the concept of a healthy endometrial microbial composition is still not settled [12,15,16,20,21]. In this model, the endometrial cavity is typically described as being composed of Lactobacillus followed by genera such as Gardnerella, Streptococcus, Bifidobacterium and Prevotella [22,23,24,25]. Lactobacillus-dominant communities are often considered more compatible with reproductive homeostasis [22,23,24,25]. Thus, several investigators have proposed to define endometrial profiles as Lactobacillus-dominant or non-Lactobacillus-dominant, as is done conceptually in vaginal microbiome studies [23,26]. Lactobacillus-dominant communities promote low vaginal pH, barrier integrity, pathogen inhibition, and balanced immune crosstalk, thereby maintaining colonization resistance and mucosal homeostasis. The female reproductive tract is shown as a hormonally responsive mucosal ecosystem in which epithelial barrier integrity, cervical mucus, immune surveillance, and microbial communities contribute to tissue homeostasis. In the lower tract, Lactobacillus-dominant communities are associated with low vaginal pH, pathogen inhibition, and balanced immune crosstalk. Toward the endometrium, microbial biomass becomes sparse, and eubiosis should be interpreted in functional terms—controlled inflammatory tone, barrier integrity, and immune balance—rather than as the dominance of a single taxon. The inset illustrates dysbiosis-associated barrier disruption, aberrant immune activation, and persistent inflammation. as shown in Figure 1.

Figure 1 is intended as a conceptual representation, in the endometrial compartment, microbial presence should be interpreted as a low-biomass microbial signal or putative community, since DNA detection does not establish viable resident microorganisms.

Vaginal and endometrial compartments should be discussed separately. They differ substantially along several axes: (1) microbial biomass—the vagina harbors a dense microbial community, whereas the endometrial cavity is regarded as a markedly lower-biomass niche, with reports suggesting 10^2^–10^4^ fewer bacteria than in the vagina [24]; (2) pH—the healthy vagina is acidic (typically pH 3.8–4.5), whereas the uterine cavity has been reported to be near neutral to mildly alkaline [27,28]; (3) immune environment—the endometrium contains specialised uterine natural killer (uNK) cells and is hormonally regulated for embryo tolerance [29]; and (4) sampling methods and contamination risks—vaginal swabs are minimally invasive, whereas endometrial samples require transcervical access with non-trivial contamination risk.

Endometrial eubiosis should be interpreted in functional rather than strictly taxonomic terms, with greater emphasis on barrier integrity, immune balance, and controlled inflammatory signaling than on the absolute dominance of any single genus [30,31,32,33,34].

### 3.2. Determinants of Microbial Variability in the Uterus

The composition of the endometrial microbiota is influenced by a wide range of host-related and methodological factors. Host-related confounders include: age, menopausal status, body mass index (BMI), obesity and metabolic syndrome, type 2 diabetes, hormone replacement therapy, hormonal treatments for endometriosis (combined oral contraceptives, progestins, GnRH analogues), recent or ongoing antibiotic exposure, probiotic or prebiotic use, probiotic or prebiotic use, diet (including dietary pattern and fiber intake), smoking status, menstrual-cycle phase, sexual activity, vaginal pH, and underlying gynaecological pathology (polyps, fibroids, hyperplasia). Methodological confounders include: sampling route (vaginal swab, cervical swab, endometrial biopsy, hysterectomy-derived tissue, endometrial lavage), contamination control, DNA extraction method, sequencing platform, targeted 16S rRNA hypervariable region, bioinformatic pipeline, and reference database. Critical confounders in endometrial cancer studies additionally include histologic subtype, tumour grade, surgical sampling, pre-operative antibiotic prophylaxis, and prior therapy. Most published studies do not adjust for the full set of confounders, which substantially limits the inferential strength of reported microbial associations [21,26,35,36,37,38,39,40].

### 3.3. Endometrial Eubiosis and Microbial Homeostasis

Endometrial eubiosis may be defined as a state of controlled microbial coexistence compatible with tissue function, reproductive competence, and limited inflammation. In this low-biomass environment, the objective of host defense is not complete sterility, but preservation of epithelial integrity, rapid containment of potentially harmful microbes, and avoidance of inappropriate inflammatory activation. For this reason, future definitions of a healthy endometrial microbiota will likely need to integrate functional parameters, including host transcriptional response, epithelial barrier status, inflammatory tone, and microbial metabolite activity [1,41,42].

### 3.4. Immune Regulation in the Endometrial Niche

The uterus is a specialized mucosal organ that has to protect against infection but also maintain tolerance for the implantation of the embryo and subsequent pregnancy [1,43,44,45,46]. The endometrial immune milieu is mainly composed of uterine natural killer (uNK) cells, macrophages, dendritic cells, neutrophils, mast cells and T lymphocytes; B cells and plasma cells are less involved [1,47,48,49,50]. This immunological architecture is especially active during the secretory phase and early pregnancy, when the tissue needs to balance surveillance, repair, angiogenesis and tolerance.

The immunological balance is closely linked to microbiota. Dysbiosis can disturb immune homeostasis and drive chronic inflammation, while commensal microbes can maintain epithelial integrity and regulated immunological activation [1,4,51,52,53]. In this context, the endometrial microbiota should not be regarded as a passive ecological bystander but as an active participant in local immunity.

### 3.5. Toll-like Receptors (TLRs) and Microbial Recognition in the Endometrium

A key mechanism linking microorganisms to uterine immunity is the expression of TLRs on endometrial epithelial cells and resident immune cells [42,54,55,56,57,58,59]. TLRs are pattern-recognition receptors that recognize bacterial lipopolysaccharides (LPS), lipoproteins, peptidoglycans and microbial nucleic acids and can trigger signaling cascades to activate NF-κB and induce cytokines, chemokines and antimicrobial agents [58,60,61,62,63]. Expression of several TLRs has been identified in the human endometrium and the abundance of these TLRs appears to vary depending on the phase of the menstrual cycle, the hormonal status and the tissue condition [41,55,56,57,61]. In this context, microbial composition matters not only because of which taxa are present, but also because of the quality and persistence of the host signaling they may induce [41,57,63,64].

### 3.6. Clinical Relevance and Pathophysiological Implications

Uterine microbiota may have a role in fertility, implantation, chronic inflammation, gynecological diseases and endometrial carcinogenesis [4,20,24,25,51,65,66]. In reproductive medicine, dysbiotic communities are associated with recurrent implantation failure and poor reproductive outcomes, whereas Lactobacillus-dominant endometrial profiles are generally associated with improved implantation and pregnancy outcomes [24,25,26,67]. Increased microbial diversity and enrichment of potentially pathogenic species have been linked to immune homeostasis disruption and chronic inflammation in conditions such as pelvic inflammatory disease and chronic endometritis [21,68,69].

In oncology, dysbiosis of the uterine environment has been associated with endometrial cancer and premalignant conditions, although causality is still unknown. Commonly reported changes include a decrease in Lactobacillus and an increase in the abundance of pro-inflammatory and anaerobic taxa such as Atopobium, Porphyromonas, Prevotella, Peptoniphilus and Anaerococcus [51,66,70]. These alterations support the hypothesisthat the uterine microbiota is a biologically relevant component of endometrial pathophysiology rather than merely a passive biomarker, which could lead to a pro-inflammatory microenvironment, impaired epithelial integrity and altered local immune responses. Existing evidence is consistent with the model of a dynamic, hormonally sensitive, immunologically integrated ecosystem of endometrial and uterine bacteria. Although the exact boundaries of eubiosis remain unclear, the microbial composition, host immunity, endocrine cycle, and tissue function are closely related [21,25,36,37].

The present review distinguishes three distinct research questions that should not be conflated: (1) what microbial alterations characterise endometriosis; (2) what microbial alterations characterise endometrial cancer; and (3) whether endometriosis-associated dysbiosis directly contributes to endometrial cancer development. Existing evidence supports questions (1) and (2) with moderate reproducibility, whereas direct human evidence supporting question (3) is currently lacking. The third question is addressed here as a testable hypothesis rather than as an established causal relationship.

## 4. Gut Microbiota and Systemic Regulation

Beyond the reproductive tract, gut microbiota is increasingly recognized as a systemic regulator of endocrine, metabolic, immune, and epithelial homeostasis. Rather than acting exclusively within the intestinal lumen, the gut microbial community influences distant organs through a broad repertoire of metabolites, enzymatic transformations, and host–microbe signaling pathways. In this sense, the gut microbiota may be conceptualized as a virtual endocrine–immunometabolism organ, capable of modulating nutrient processing, epithelial turnover, neuroendocrine communication, immune education, and steroid hormone availability. This systemic perspective has become especially important in contemporary biomedical research, because it places the intestinal microbiome at the centre of host physiology rather than at its periphery [71,72].

Among the best-characterized mediators of gut microbiota–host communication are short-chain fatty acids (SCFAs), especially acetate, propionate, and butyrate, which are produced through the fermentation of dietary fiber by microorganisms. These metabolites are not merely end-products of microbial metabolism; they function as active signaling molecules that help maintain epithelial integrity, support mucus production, reinforce tight-junction architecture, and shape both local and systemic immune responses. SCFAs also act through G-protein-coupled receptors and epigenetic mechanisms, including histone deacetylase inhibition, thereby influencing inflammatory tone, leukocyte differentiation, and immune tolerance. Through these actions, SCFAs participate in the preservation of gut barrier function and in the limitation of systemic exposure to luminal antigens and endotoxins [72,73].

In parallel, the gut microbiota contributes to the transformation of bile acids and the control of intestinal permeability, both of which have effects outside of the gastrointestinal system.

Microbial deconjugation and conversion of primary into secondary bile acids alter host metabolic signaling through pathways such as the Farnesoid X receptor (FXR) and Takeda G protein-coupled receptor 5 (TGR5), while disruption of barrier integrity facilitates translocation of LPS and other microbe-associated molecular patterns into the circulation.

This may result in a prolonged low-grade inflammatory state marked by activation of innate immune pathways, cytokine production, and altered immune-cell behaviour. Based on this, the gut microbiota is now recognised as a crucial interface that connects metabolism, digestion, systemic inflammation, and epithelial defence [71,74].

A major concept linking the gut microbiota to systemic steroid regulation is the estrobolome, generally defined as the collection of enteric microbial genes and enzymes capable of metabolizing estrogens and thereby influencing their bioavailability. This concept emerged from the broader recognition that intestinal microorganisms contribute not only to nutrient metabolism but also to the enterohepatic circulation of steroid hormones. After hepatic phase II metabolism, estrogens are commonly conjugated, particularly as glucuronides and sulphates, and secreted into the bile for intestinal excretion. Within the estrobolome, β-glucuronidase is the most well-characterized and biologically relevant enzyme. Bacterial β-glucuronidases can cleave glucuronic acid residues from conjugated estrogen metabolites, regenerating the active, deconjugated forms of estrone and estradiol, and increasing the pool of estrogens available for reabsorption. This enzymatic step is central to the concept of enterohepatic estrogen recycling. Although β-glucuronidase has received the greatest attention, the estrobolome should not be reduced to a single enzyme; rather, it reflects a broader functional capacity distributed across multiple microbial taxa and potentially involving additional deconjugating and transforming activities. Consequently, the estrobolome is better understood as a community-level metabolic property than as the activity of one microorganism alone [75,76,77].

The biologic importance of the estrobolome lies in the fact that even modest shifts in microbial composition or enzymatic output may alter systemic estrogen exposure. If microbial deconjugation is enhanced, a greater proportion of estrogen metabolites may escape fecal elimination and re-enter the circulation; conversely, reduced deconjugation may favor estrogen excretion. The overall result depends on a complex interaction between hepatic conjugation, microbial enzyme abundance, intestinal transit, diet, fiber availability, antibiotic exposure, age, body composition, and the structure of the microbial community itself. Thus, the estrobolome should be viewed as a functional axis at the intersection of host metabolism and microbial ecology rather than as a fixed or uniform biological entity [75,77].

Importantly, the estrobolome is not merely a theoretical construct. In a cross-sectional human study, Flores et al. showed that fecal microbiome richness and diversity were significantly associated with non-ovarian estrogen levels and estrogen metabolites, supporting a link between gut microbial ecology and systemic estrogen balance. Specifically, fecal β-glucuronidase activity was associated with urinary estrone [71], suggesting a role for microbial enzymatic function in the in vivo enterohepatic recirculation of estrogens [71]. This was further supported by the biochemical studies of Ervin et al. who directly demonstrated that several human gut microbial β-glucuronidase enzymes can deconjugate estrone-3-glucuronide and estradiol-17-glucuronide, thereby reactivating biologically relevant estrogens from their inactive conjugated forms [75]. Together, these studies provide both physiologic and mechanistic support for the estrobolome model [75,76,77,78,79,80,81].

## 5. Methodological Considerations and Contamination Risks in Low-Biomass Microbiome Studies

The endometrial cavity is a low-biomass microbial niche, in which bacterial DNA concentrations are typically orders of magnitude lower than in the vagina or the gut. This imposes substantial methodological constraints that directly affect the interpretation of microbiome studies in both endometriosis and endometrial cancer. Several issues deserve explicit attention.

### 5.1. Sampling Contamination

Endometrial tissue is most commonly obtained by transcervical biopsy, hysteroscopic sampling, or curettage. Each route carries a non-negligible risk of contamination by vaginal or cervical microbiota, particularly when catheters or brushes pass through the cervix. Surgical (hysterectomy-derived) specimens generally offer cleaner access but remain exposed to skin and operating-room microbial DNA. Cross-sectional designs cannot distinguish true upper-tract colonisation from procedural contamination.

### 5.2. Reagent Contaminants

Commercial DNA extraction kits, PCR reagents, and laboratory consumables frequently contain trace bacterial DNA. When endogenous microbial biomass is very low, these contaminants may dominate the sequencing output unless systematically identified and removed.

### 5.3. Negative and Positive Controls

Rigorous low-biomass studies include DNA extraction blanks, PCR no-template controls, and positive controls (mock communities) processed in parallel with every batch. The absence of such controls undermines the reliability of reported taxa.

### 5.4. Bacterial Load Quantification

Quantitative PCR (qPCR) targeting the 16S rRNA gene, or digital PCR, should be used to estimate total bacterial load per sample. Low-biomass samples with undetectable bacterial DNA require cautious interpretation, since any signal detected may be artefactually dominated by contamination.

### 5.5. Decontamination Pipelines

Bioinformatic tools and prevalence-based filtering against identified kit-ome contaminants can reduce false-positive taxa but cannot fully substitute for clean laboratory practice.

### 5.6. DNA Detection Versus Live Microorganisms

Most existing studies rely on 16S rRNA gene sequencing or shotgun metagenomics, which detect microbial DNA but do not discriminate between live, viable microorganisms, dormant or dead cells, and free DNA. The demonstration of a transcriptionally or metabolically active microbial community requires meta-transcriptomics, cultivation, or microscopy-based confirmation, which is rarely performed.

For these reasons, microbial signals reported in the uterine compartment should be interpreted as hypotheses requiring replication and functional validation rather than as definitive evidence of a resident and biologically active endometrial microbiome.

## 6. Endometriosis-Associated Dysbiosis

**Figure 2:**
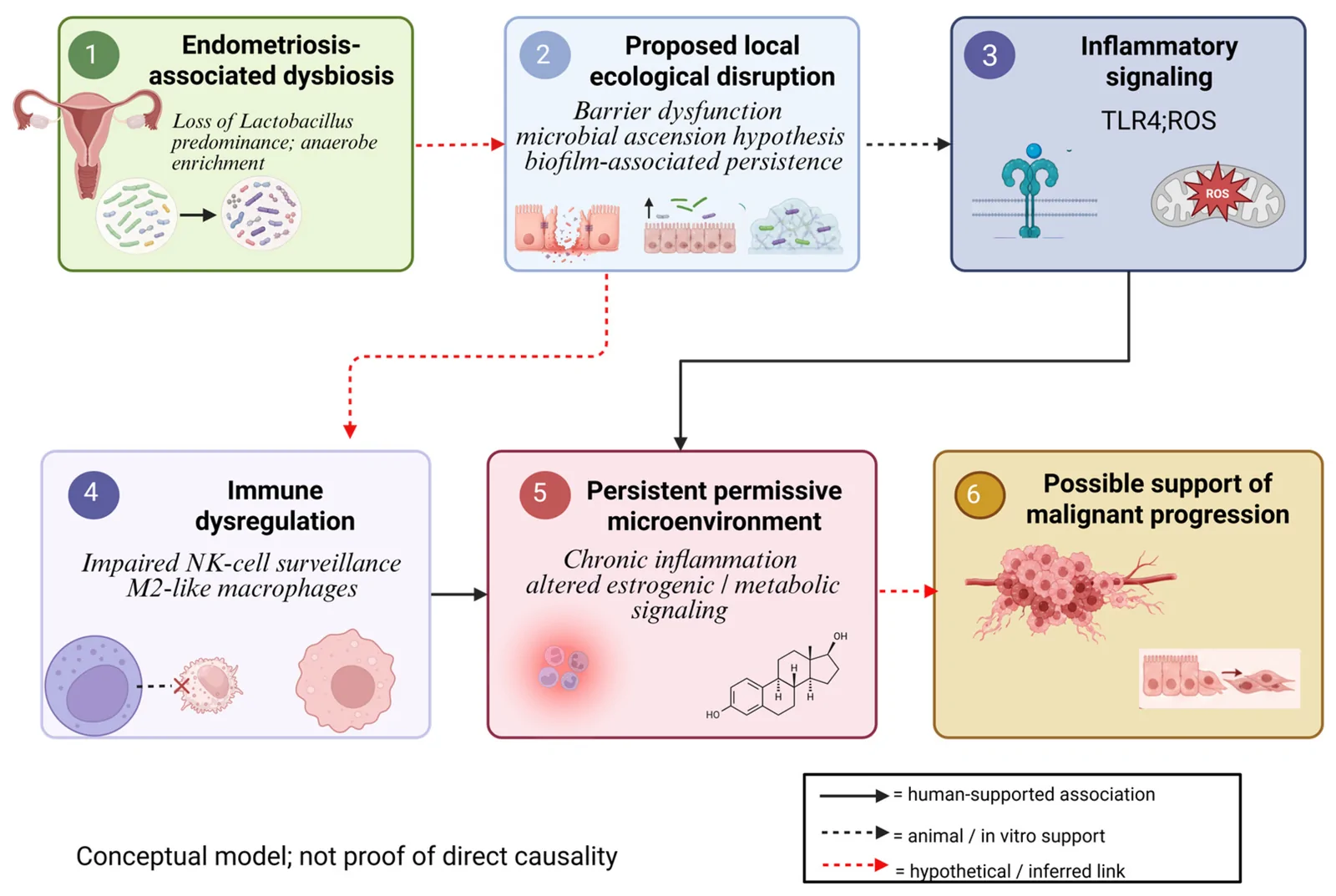
Endometriosis-associated dysbiosis: inflammatory and pro-tumorigenic mechanisms.

Although no single reproducible dysbiosis signature has been identified, current research indicates that endometriosis is linked to changes in both gut and reproductive tract microbial ecosystems. Across cohorts, the most recurrent observations include loss of Lactobacillus predominance, enrichment of anaerobic or pro-inflammatory taxa, and substantial heterogeneity according to sample type and analytic method. Thus, endometriosis-associated dysbiosis is best interpreted as ecological instability with potential functional consequences rather than as a fixed microbial fingerprint [80,81]. Loss of Lactobacillus dominance and enrichment of anaerobic bacteria may impair epithelial barrier function and reshape the endometrial immune niche toward immune evasion. M2-like macrophages, dysfunctional NK cells, suppressed CD8+ T-cell activity, and tolerogenic metabolic signaling may collectively support the persistence of altered clones and tumor progression. These findings are illustrated in Figure 2.

Figure 2 illustrates four intertwined mechanisms. Microbial ascension from the lower genital tract is currently supported by bacterial contamination–hypothesis evidence in peritoneal fluid and menstrual blood [82]. Repeated influx of LPS and other microbial products can drive oxidative stress via iron-dependent ROS generation within ectopic lesions, particularly in the context of cyclic hemorrhage [83,84]. Microbial metabolites and inflammatory mediators can sensitize nociceptive afferents, plausibly contributing to pain presentation. Finally, in analogous cancer contexts, certain bacterial toxins (e.g., colibactin produced by pks-positive *E. coli*) can induce double-strand DNA breaks, whether analogous genotoxic mechanisms operate in the endometrial niche remains hypothetical.

### 6.1. Gut Dysbiosis in Endometriosis

Gut dysbiosis in endometriosis appears to involve compositional instability rather than a universal taxonomic pattern. Studies with sample sizes under 30 participants per group suggest altered diversity and changes in specific bacterial groups, whereas cohorts with sample sizes above 100 participants per group indicate that such differences may be subtle, context-dependent, or influenced by confounding factors [85,86]. Even so, mechanistic links remain highly plausible.

First, gut dysbiosis may contribute to systemic inflammation by disrupting the intestinal barrier and promoting translocation of endotoxins such as LPS. This can activate TLR4- and NF-κB-related pathways and increase pro-inflammatory mediators that indirectly support lesion implantation and survival. Specifically, the enrichment of Gram-negative families such as Enterobacteriaceae in experimental models, has been reported to promote the proliferation of endometriotic stromal cells; whether this contributes to a pro-carcinogenic microenvironment in humans remains hypothetical [30].

Second, dysbiosis may influence estrogen metabolism through altered β-glucuronidase activity, thereby enhancing enterohepatic estrogen recirculation and reinforcing the estrogen-dependent nature of endometriosis. This estrobolome disruption has been hypothesised to contribute to a state of relative hyperestrogenism. Whether chronic hyperestrogenism in this context is directly associated with malignant transformation remains uncertain, including via overactivation of PI3K/AKT signalling, is supported primarily by data from endometriosis-associated ovarian (rather than endometrial) carcinogenesis and by experimental models [87].

Third, microbiota-derived metabolites may affect nociceptive pathways, suggesting a possible connection with pain and disease severity, although robust human evidence is still limited [81,85,86,88,89]. Beyond pain, the reduction in metabolic diversity can lead to a decrease in protective metabolites, failing to inhibit the oxidative stress that contributes to DNA damage within the lesions.

### 6.2. Endometrial and Uterine Microbiota in Endometriosis

Reproductive tract dysbiosis in endometriosis has been described at the vaginal, cervical, and endometrial levels. Although consistent global diversity changes are not always found, multiple studies report taxon-level shifts in women with endometriosis, particularly in cervical and endometrial samples. These alterations suggest that the local reproductive microenvironment is modified in association with disease presence and chronic inflammation [81,90,91]. Specifically, the shift from a Lactobacillus-dominant environment to one enriched with opportunistic pathogens (e.g., Actinobacteria, Enterobacteriaceae) may compromise the mucosal barrier and alter local pH, favoring a niche that supports cellular atypia. Crucially, most existing studies report site-specific findings (vaginal, cervical, or endometrial) that should not be extrapolated across compartments. Whether the reported shifts at the vaginal or cervical level correlate with endometrial dysbiosis, in endometriosis remains to be established.

One influential explanatory model is the bacterial contamination or microbial translocation hypothesis, according to which bacteria or endotoxins may ascend from the lower genital tract or contaminate menstrual reflux, thereby reaching the uterine cavity and peritoneal space. In this model, microbial products such as LPS activate TLR4-dependent inflammatory pathways, promoting lesion establishment and persistence [82]. Crucially, this chronic activation of TLR4-dependent signaling not only support lesion survival but also induces the secretion of pro-angiogenic factors and cytokines like IL-6, which are implicated in the early stages of malignant transformation and epithelial–mesenchymal transition (EMT) [92]. In parallel, loss of Lactobacillus dominance and possible biofilm formation may help sustain local inflammation, reduce immune clearance, and favor chronic mucosal dysfunction [4,81,82]. The presence of these polymicrobial biofilms has been proposed to act as a persistent source of local genotoxic stress. In other cancer types, certain bacterial toxins (e.g., colibactin produced by pks-positive E. coli) have been shown to induce DNA damage; however, whether analogous mechanisms operate in the endometrial niche remains unknown [87].

### 6.3. Dysbiosis, Chronic Inflammation and Lesion Persistence

Dysbiosis may exacerbate the chronic inflammatory milieu that characterizes endometriosis (see Section 3.5) [88,89,93]. Moreover, the persistent activation of these cytokines, particularly IL-6 and TNF-α, has been proposed, primarily on the basis of in vitro and animal studies, to contribute to oncogenic signalling pathways such as STAT3. Whether this pathway is directly implicated in the initiation of endometrial carcinogenesis in humans remains to be established [87].

At the cellular level, lesion persistence is associated with increased recruitment or activation of macrophages and neutrophils, altered T-cell responses, and impaired NK-cell cytotoxicity. Rather than efficiently clearing ectopic endometrial cells, the immune system appears functionally reprogrammed toward a permissive state in which inflammation persists, but elimination fails. This combination of immune activation and defective clearance is central to the chronicity of endometriosis [1,93,94]. Dysbiosis-induced shifts in macrophage polarisation towards an M2-like phenotype have been reported in preclinical models. These macrophages secrete growth factors and matrix metalloproteinases (MMPs) that facilitate tissue remodelling and invasion—processes relevant to endometriosis progression. Whether dysbiosis-induced M2 polarisation also contributes to malignant transformation in humans remains speculative. Similarly, NK-cell dysfunction has been documented in endometriosis and other inflammatory conditions; however, whether microbiota alterations directly impair NK-cell surveillance in the endometrial niche remains unclear [95].

### 6.4. Bidirectional Interactions Between Endometriosis and Microbiome

Taken together, current data support a bidirectional model in which endometriosis and the microbiome reinforce each other. On the one hand, endometriosis modifies the local and systemic microenvironment through cyclic bleeding, iron overload, oxidative stress, inflammatory cytokines, and altered estrogen signaling. These changes can destabilize epithelial barriers and reshape microbial communities in both the gut and the reproductive tract [81,82,96]. The accumulation of heme-derived iron within the peritoneal cavity may favour the expansion of siderophilic bacteria, which has been proposed, on the basis of in vitro data, to amplify oxidative DNA damage in ectopic endometrial cells [30].

On the other hand, a dysbiotic microenvironment may feed back to sustain endometriosis by amplifying LPS/TLR4-driven inflammation, increasing estrogen recirculation through estrobolome activity, promoting barrier dysfunction, and weakening effective immune clearance. This creates a circular pathogenic model in which dysbiosis, and endometriosis are not independent phenomena butpotentially interacting processes. Their interaction may help explain chronic inflammation, lesion persistence, and possibly progression toward malignancy in susceptible settings [80,81,88,89,96]. The transition from endometriosis to malignant transformation, when it occurs, is best documented in the ovarian compartment (clear-cell and endometrioid carcinomas). The synergy between hyperestrogenism (maintained in part by a disrupted estrobolome) and the genotoxic stress that may be induced by dysbiotic metabolites has been hypothesised to facilitate the acquisition of somatic mutations in ARID1A, PIK3CA, and KRAS in ovarian endometriosis-associated carcinomas. Oxidative iron injury arising from heme accumulation within ectopic endometrial lesions has been documented primarily in the context of endometriosis-associated ovarian carcinoma; whether analogous mechanisms operate in the uterine endometrium remains speculative. Whether, analogous processes operate in the endometrium is not currently supported by direct evidence and their role, in uterine endometrial carcinogenesis remains speculative [87].

Table 1 summarizes the principal original studies evaluating microbiota alterations in endometriosis, highlighting the heterogeneity of sample sources, sequencing strategies, and disease-associated microbial signatures across the gut, peritoneal fluid, and the lower and upper reproductive tracts. Taxa listed in Table 1 should be interpreted as study-specific associations rather than validated biomarkers. The included studies are predominantly small, cross-sectional, and heterogeneous in sampling site, anatomical compartment, sequencing platform, and analytical pipeline, which precludes derivation of a reproducible disease signature from the current literature.

## 7. Pathogenesis of Endometrial Cancer

In high-income nations, endometrial cancer is the most prevalent gynaecologic cancer, and its prevalence is still rising worldwide, although part of this increase may reflect population aging and changes in hysterectomy rates. The most substantial driver appears to be the growing prevalence of obesity, insulin resistance, and other metabolic disorders. Endometrial cancer occurs predominantly in postmenopausal women; however, a clinically relevant proportion of cases arises in younger patients, particularly in the setting of endocrine dysfunction, chronic anovulation, obesity, and inherited cancer susceptibility syndromes.

Established clinical risk factors include advanced age, menopausal status, endometrial hyperplasia, prolonged or unopposed estrogen exposure, tamoxifen use, low parity, Lynch syndrome, diabetes, hypertension, metabolic syndrome, and obesity. Importantly, endometrial cancer also displays significant disparities in incidence, histologic distribution, and survival across racial and ethnic groups, indicating that tumour biology intersects with environmental, metabolic, and socioeconomic determinants of health [5,102,103,104,105,106,107,108,109,110,111,112,113,114,115,116,117,118,119].

The molecular era has greatly improved our understanding of endometrial carcinogenesis. The Cancer Genome Atlas (TCGA) identified four molecular subgroups of endometrial carcinoma: POLE-ultramutated, MSI-hypermutated, copy-number low, and copy-number high. These categories are based on integrative genomic analysis and are widely used in research settings.

The Proactive Molecular Risk Classifier for Endometrial cancer (ProMisE) is a clinically applicable surrogate that classifies endometrial carcinomas into POLE-mutated, MMR-deficient, p53-abnormal, and NSMP categories using immunohistochemistry and targeted sequencing. ProMisE correlates substantially but not perfectly with TCGA categories and has the practical advantage of being applicable to formalin-fixed paraffin-embedded tissue. Two classification systems should not be presented as identical or interchangeable. This review uses TCGA terminology when referring to genomic studies and ProMisE terminology when referring to clinically annotated cohorts. ProMisE-derived categories are now increasingly integrated into routine pathology reporting and treatment planning [120,121].

At the mechanistic level, endometrial cancer is strongly driven by dysregulation of the PI3K/AKT/mTOR pathway and related proliferative signaling networks. Aberrant PI3K/AKT activation promotes cell survival, growth, metabolic adaptation, and resistance to apoptosis and is particularly relevant in endometrioid carcinomas, in which somatic alterations in PTEN, PIK3CA, KRAS, and ARID1A are well documented. ARID1A and PIK3CA mutations are also strongly associated with endometriosis-associated ovarian clear-cell and endometrioid carcinomas, and data from these ovarian malignancies cannot be directly extrapolated to endometrial carcinogenesis [83,106,122,123,124,125].

### 7.1. Hormonal and Endocrine Mechanism

Hormonal imbalance remains at the core of the pathophysiology of endometrial cancer, especially in endometrioid disease. Through both genomic and non-genomic mechanisms, estrogen promotes endometrial proliferation. Estrogen receptor signaling regulates transcriptional programs involved in cell cycle progression and differentiation at the genomic level. At the non-genomic level, estrogen rapidly activates proliferative cascades, such as PI3K/AKT and mitogen-activated protein kinase/Extracellular signal-regulated kinase (MAPK/ERK), thus reinforcing growth-promoting signals. Without, or with inadequate or functionally ineffective progesterone, this proliferative estrogenic stimulus is not adequately antagonized, allowing progression from endometrial hyperplasia to atypia and ultimately carcinoma. Progesterone normally exerts antiproliferative, differentiating, and anti-inflammatory effects and its loss or resistance has important biological and therapeutic implications. Therefore, endometrial cancer should be considered not only an estrogen-dependent tumor but also a tumor that arises from disrupted endocrine balance and steroid receptor signaling [126,127,128,129].

### 7.2. Obesity, Diabetes, Hypertension and Metabolic Reprogramming

Obesity, diabetes, and related metabolic dysfunction are major contributors to endometrial carcinogenesis because they integrate aromatase-driven estrogen production, chronic low-grade inflammation, adipokine imbalance, insulin resistance, oxidative stress, and growth factor signaling. These processes are also relevant to microbiome-oriented models, since gut dysbiosis may amplify metabolic inflammation, alter barrier function, and reinforce endocrine disequilibrium. Accordingly, metabolic risk should be viewed not as a parallel pathway separate from the microbiome but as part of the same endocrine–immunometabolism network [5,106,116,117,118].

### 7.3. Microbiome and Endometrial Carcinogenesis

The microbiome has emerged as a plausible participant in endometrial carcinogenesis because both gut and reproductive tract microbial communities are influenced by obesity, menopause, inflammation, endocrine disruption, and metabolic disease. Rather than replacing established endocrine and molecular models of endometrial cancer, microbiome-oriented interpretations help integrate them within a broader systems-biology framework centered on barrier integrity, inflammatory tone, immune regulation, microbial metabolites, and steroid hormone handling.

**Figure 3:**
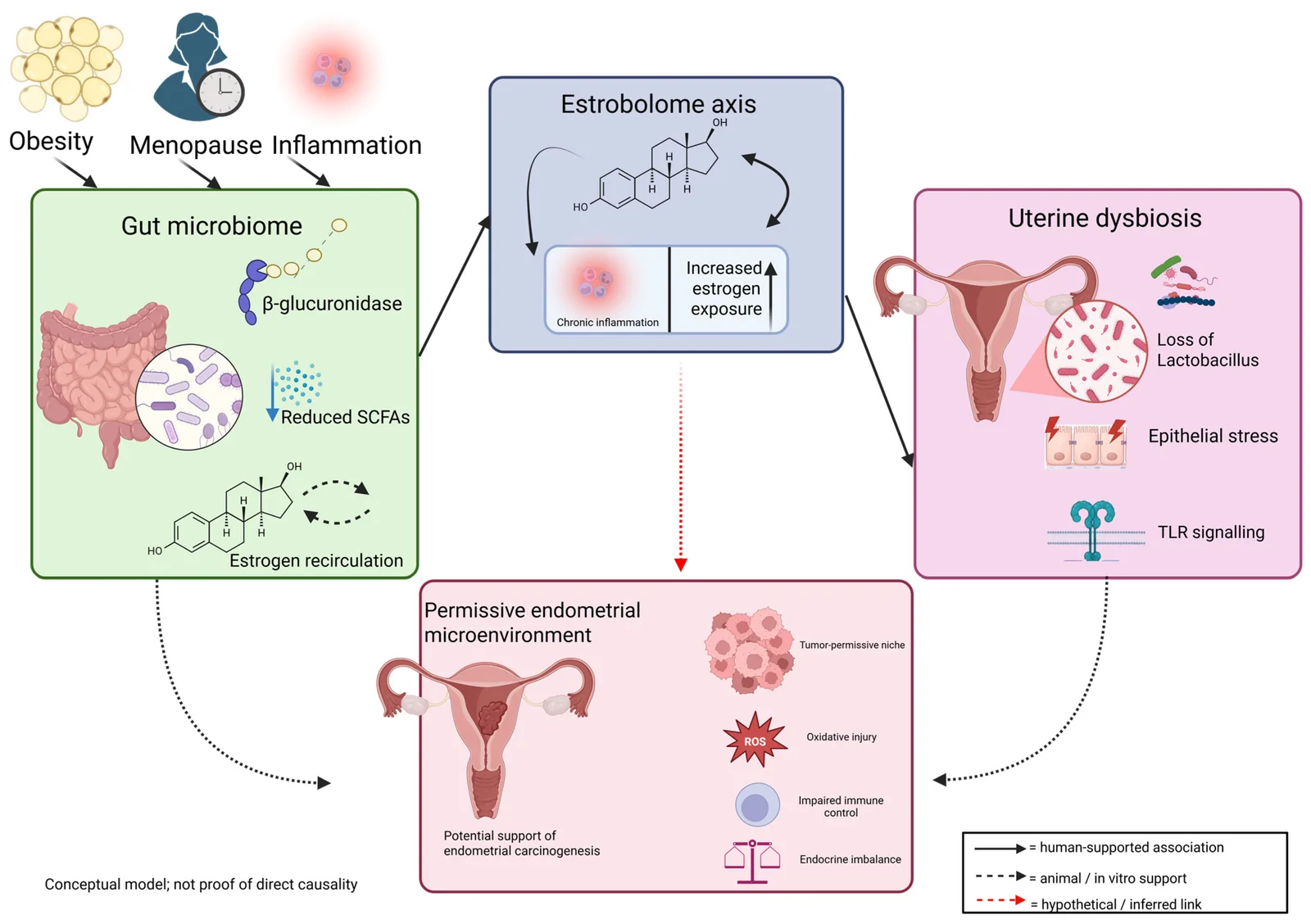
Gut–uterus estrobolome axis in endometrial carcinogenesis.

The estrobolome remains one of the most compelling links in this framework. By modulating estrogen deconjugation and enterohepatic recirculation, gut microbial enzymes may influence systemic hormonal exposure, while local reproductive tract dysbiosis may intensify inflammatory signaling through enrichment of anaerobic and pro-inflammatory taxa. In parallel, altered microbial metabolites, including reduced SCFA availability and other bioactive compounds, may contribute to chronic inflammation, oxidative stress, and epigenetic plasticity, thereby reinforcing a tumor-permissive endometrial microenvironment [36,130,131,132,133,134]. The local uterine microbiota may also directly contribute to carcinogenesis. Endometrial dysbiosis has been correlated with loss of Lactobacillus dominance and relative enrichment of taxa such as Prevotella, Porphyromonas, Peptoniphilus and Anaerococcus in endometrial cancer cohorts. These organisms may stimulate inflammatory signaling, alter local immune tone and may promote epithelial stress. In addition, microbial pattern recognition through TLRs may serve as a bridge between dysbiosis and tumor-promoting inflammation, thereby linking microbial imbalance with stromal activation, immune evasion, and disease progression [6,36,130,135]. Figure 3 illustrates the gut–uterus estrobolome axis in endometrial carcinogenesis.

The figure depicts an evidence-graded conceptual model in which the estrobolome serves as a central biochemical link between gut dysbiosis, altered estrogen metabolism, and endometrial microenvironmental change. Through β-glucuronidase-mediated deconjugation of estrogen metabolites, microbial activity may influence enterohepatic estrogen recirculation and systemic estrogen exposure. Gut- and uterine-associated dysbiosis may further reinforce inflammatory and metabolic stress, thereby contributing to a permissive endometrial environment. The proposed association is one of biologic facilitation rather than straightforward direct causality.

### 7.4. Microbial Dysbiosis Related to Endometrial Cancer

Over the last decade, the relationship between the microbiome and endometrial cancer has evolved from an exploratory hypothesis into a distinct research field within endometrial carcinogenesis. The current literature no longer supports the historical assumption that the uterine cavity is sterile; instead, it suggests that the endometrium harbors low-biomass microbial communities whose ecological disruption may be linked to malignant transformation. In narrative terms, the evidence has progressed in a recognizable sequence: first, descriptive cross-sectional studies identified compositional differences between benign and malignant endometrial states—most consistently reduction in Lactobacillus abundance and relative enrichment of Porphyromonas, Peptostreptococcus, Atopobium, Anaerococcus, and Prevotella [10] potentially relevant biological mechanisms include: TLR2/4-mediated inflammatory activation by bacterial products, β-glucuronidase-driven modulation of local oestrogen metabolism, and genotoxic stress from bacterial metabolites—although each of these mechanisms currently rests on preclinical or extrapolated evidence; second, validation studies have attempted to connect these differences with host risk factors including postmenopausal status, obesity, and vaginal pH; and third, integrative and mechanistic studies have begun to link microbial dysbiosis with inflammation, metabolism, immune escape, and tumour progression, though direct human causal evidence remains limited [7,122,123].

The literature on microbial dysbiosis in endometrial cancer has evolved from descriptive taxonomic surveys toward more integrative models linking dysbiosis to inflammation, endocrine disturbance, immune escape, and tumor biology. Foundational studies identified tract-wide ecological shifts associated with endometrial cancer, particularly reduced Lactobacillus abundance and enrichment of anaerobic taxa such as Atopobium, Porphyromonas, Peptoniphilus, Prevotella, and Anaerococcus. Later work connected these findings to clinical risk factors including postmenopausal status, obesity, and elevated vaginal pH. Subsequent studies broadened the field by integrating transcriptomic, metatranscriptomic, lavage-based, organoid, and multi-site approaches. These investigations suggest that endometrial cancer dysbiosis is not confined to the uterus but may involve a broader disturbed mucosal network affecting the vagina, cervix, endometrium, and rectal niche. Reported associations with inflammatory, coagulation-related, metabolic, and immune-tolerance pathways support the biological plausibility of microbial involvement without proving causality.

At the same time, the field remains methodologically fragile. The endometrium is a low-biomass niche, and reported microbial signals are vulnerable to contamination, sampling bias, sequencing variability, and cohort heterogeneity. Thus, the most robust conclusion at present is not the existence of a single oncobacterial agent, but the recurrence of ecological destabilization characterized by lactobacilli depletion, increased heterogeneity, enrichment of mixed anaerobic taxa, and growing links to inflammatory and immunometabolic remodeling [83,124,125,126,127,128,136,137,138,139,140]. Table 2 summarizes the principal original studies evaluating microbiota alterations in endometrial cancer, highlighting the heterogeneity of sample sources, sequencing strategies, and disease-associated microbial signatures across uterine, cervicovaginal, and rectal compartments.

The gut–uterus axis provides a useful framework through which gut dysbiosis may influence endometrial carcinogenesis. The estrobolome offers the clearest biochemical mechanism since microbial β-glucuronidase activity may increase enterohepatic estrogen recirculation and amplify hormonal exposure in already susceptible hosts, particularly obese and postmenopausal women. At the same time, reduced SCFA production, increased intestinal permeability, and chronic endotoxin exposure may reinforce low-grade inflammation, metabolic dysfunction, and cytokine signaling relevant to endometrial tumor biology.

These systemic alterations may also have local correlates within the reproductive tract. Studies of endometrial cancer-associated dysbiosis have repeatedly described reduced lactobacilli dominance, enrichment of anaerobic taxa, and links to inflammatory, transcriptomic, and immune tolerance pathways. Taken together, current evidence supports a model in which endocrine dysregulation, chronic inflammation, metabolic imbalance, and local reproductive tract dysbiosis converge on a shared tumor-permissive program; however, this model remains biologically plausible rather than definitively proven [6,83,122,123,124,125,136,137,138,139,140]. Taxa and functional signatures listed in Table 2 should be interpreted as study-specific associations rather than reproducible disease signatures or validated biomarkers. Most available studies are small, cross-sectional, anatomically heterogeneous, and variably controlled for menopause, BMI, histologic subtype, and contamination risk. “NR” indicates not reported in the original publication.

## 8. Endometriosis-Associated Dysbiosis Implications in Endometrial Carcinogenesis

This section integrates the three questions outlined above into a mechanistic hypothesis. Importantly, direct human evidence supporting the contribution of endometriosis-associated dysbiosis to endometrial carcinogenesis remains indirect. Most of the mechanisms discussed below have been documented in endometriosis-associated ovarian carcinogenesis, in non-gynaecological malignancies, or in experimental models, and should be interpreted accordingly.

Although endometriosis is typically thought of as a benign estrogen-dependent condition, it shares a number of biologic characteristics with neoplastic processes, such as resistance to apoptosis, invasive behavior, angiogenic activation, oxidative stress, immune evasion, and persistent inflammatory signaling [83]. Within this framework, endometriosis-associated dysbiosis may represent a hypothetical link between chronic mucosal dysfunction and a permissive microenvironment for malignant transformation. Rather than representing an isolated microbial abnormality, dysbiosis appears to participate in a broader endocrine–immunometabolic disturbance that may create permissive conditions for endometrial carcinogenesis [122,123,124]. In this model, altered microbial communities do not simply coexist with disease, may plausibly contribute to lesion persistence, aberrant tissue repair, and host responses compatible with tumour promotion [127,128,152,153,154].

### 8.1. Cohort Heterogeneity and Cross-Disease Comparisons

Most endometriosis microbiome studies enrol reproductive-age women (typically 20–45 years), whereas endometrial cancer cohorts are predominantly postmenopausal (typically 55–75 years). Apparent microbial differences between these groups may therefore reflect age, menopausal status, hormonal milieu, BMI, or obesity rather than a true biological continuum from endometriosis to endometrial cancer. Comparisons across these populations should be interpreted cautiously.

**Figure 4:**
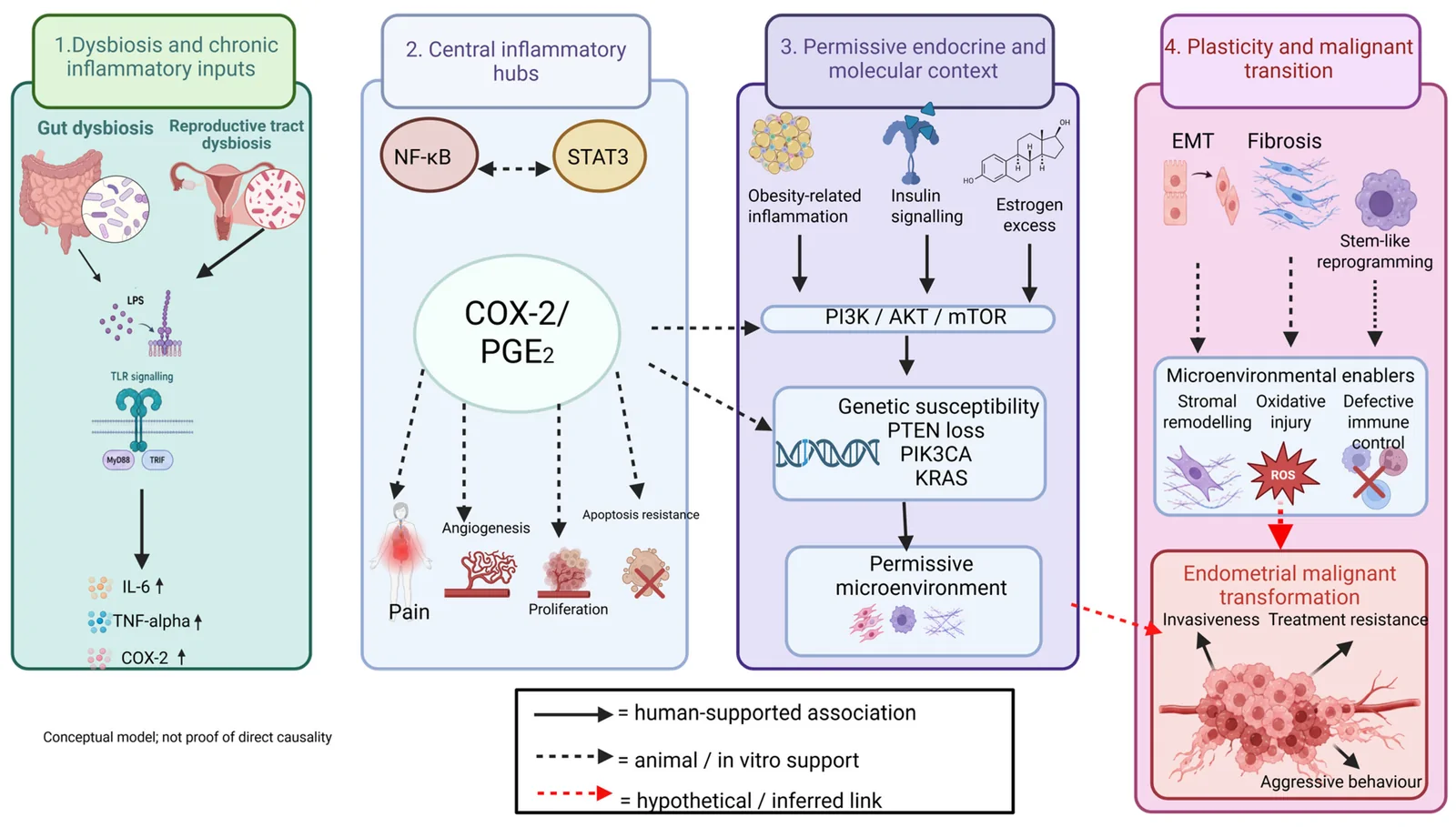
Inflammatory signalling pathways linking dysbiosis to endometrial malignant transformation.

Both endometriosis and endometrial cancer are associated with ecological instability across the female reproductive tract and, more broadly, with disturbed gut–genital tract crosstalk. This dysbiotic state is characterized by reduced Lactobacillus dominance, enrichment of anaerobic and pro-inflammatory taxa, altered metabolite production, and disturbed host–microbe signaling. Within the present section, dysbiosis is interpreted as part of a wider system-level disturbance that integrates endocrine, inflammatory, metabolic, and immunologic dysfunction, rather than as an isolated microbial phenomenon [122,123,125,136]. Inflammatory signaling pathways linking dysbiosis to endometrial malignant transformation are illustrated in Figure 4.

The figure illustrates a conceptual, evidence-graded framework in which dysbiosis-associated inflammatory stress may activate NF-κB-, STAT3-, and COX-2/PGE2-related signalling and interact with endocrine and molecular factors, including obesity-related inflammation, insulin signalling, estrogen excess, and PI3K/AKT/mTOR pathway activity. These processes may promote chronic inflammatory remodelling, altered tissue plasticity, and a permissive microenvironment. The downstream connection to malignant progression remains biologically plausible but not definitively proven in human endometrial carcinogenesis.

### 8.2. Shared Biological Mechanisms Across Endometriosis and Endometrial Cancer

One of the major mechanisms by which endometriosis-associated dysbiosis may promote carcinogenesis is the amplification of inflammatory and innate immune signaling; although this evidence is primarily derived from endometriosis-associated ovarian carcinoma, its extrapolation to endometrial carcinogenesis remains hypothetical [119,120,121,129,155]. For descriptive background on NF-κB and STAT3 signaling, COX-2/PGE2 amplification, macrophage polarization, and impaired NK-cell surveillance, see Section 6.3 and Section 6.4. The translational significance of these mechanisms for endometrial carcinogenesis is reviewed here, with emphasis on how they may converge on a permissive tissue microenvironment rather than on their descriptive characterization [120,149,150,156]. In this context, dysbiosis-driven inflammatory signaling can gradually lower the threshold for malignant transformation by maintaining epithelial stress, stromal remodeling and proliferative drive [152,153,157].

### 8.3. Endometriosis-Specific Dysbiosis Contribution to Carcinogenesis

Oxidative stress, immune dysfunction, and the endometriosis-endometrial cancer interface. Endometriosis provides a biologically relevant inflammatory and oxidative framework. Chronic inflammation, excessive prostaglandin and cytokine signalling, immune dysfunction involving macrophages and NK cells, and persistent ROS production may together support genomic instability and resistance to apoptosis. The accumulation of heme-derived iron within endometriotic lesions and the peritoneal cavity further contributes to a pro-oxidative milieu, which has been proposed—though not directly proven in the uterine endometrial context—to favour somatic mutagenesis. Although endometriosis and endometrial cancer have a less direct epidemiological association than endometriosis and ovarian cancer, the mechanistic overlap is substantial and may be particularly relevant in younger women with combined inflammatory and endocrine dysregulation [89,152,153,154].

Endometriosis-related dysbiosis can induce macrophage polarization into tumor-supportive phenotypes, impair effective immune clearance and decrease immune surveillance in the local microenvironment [122,123,125,136]. Such changes may promote a tolerant rather than immunologically vigilant niche, allowing damaged or aberrantly proliferating cells to persist. At the same time, chronic inflammatory activation and altered host–microbe signaling may disturb the balance between tissue repair and persistent injury, thereby facilitating long-term lesion survival and increasing oncogenic susceptibility [152,153,157].

In the context of endometriosis-associated dysbiosis, estrogen metabolism is another important link between dysbiosis and endometrial cancer. By modulating estrogen recirculation, altered microbial communities may contribute to chronic hyperestrogenism signaling, thereby reinforcing estrogen-dependent proliferation and progesterone resistance. When this endocrine imbalance occurs together with dysbiosis-induced inflammation and impaired immune surveillance, it may support a biological environment permissive for endometrial carcinogenesis. Thus, endometriosis-associated dysbiosis may be understood not only as an inflammatory disturbance but also as a hormonal amplifier of malignant potential [122,123,124].

The estrobolome-mediated increase in systemic estrogen exposure would, on biological grounds, be expected to be most relevant to hormone-responsive endometrioid tumors, particularly NSMP endometrioid carcinomas in the ProMisE classification. By contrast, a permissive inflammatory and immune-evasive microenvironment could, in principle, sustain the persistence of precursor lesions across several molecular subtypes, including p53-abnormal tumors.

Endometriosis-associated dysbiosis interacts with the PI3K/AKT/mTOR pathway is presented as a major survival and proliferation axis linking endometriosis-associated inflammatory remodeling with endometrial carcinogenesis. PTEN loss and activating PIK3CA and KRAS mutations are well-documented in uterine endometrioid endometrial carcinomas. By contrast, ARID1A loss is more strongly characteristic of endometriosis-associated ovarian clear-cell carcinomas. Whether endometriosis-associated dysbiosis modulates any of these molecular pathways in the uterine endometrium remains unestablished. The present observations are most consistent with the interpretation that dysbiosis, when present, may amplify a permissive microenvironment rather than directly initiating malignant transformation [105,158,159].

Endometriosis-associated environments are characterized by sustained inflammatory signaling and oxidative injury, and the accumulation of heme-derived iron may further promote conditions favorable to pathogenic microbial expansion and oxidative DNA damage. In parallel, epithelial–mesenchymal transition, fibrotic remodeling, and stem-like reprogramming are presented as processes that contribute to invasiveness and to the emergence of a pro-oncogenic niche. Within this framework, dysbiosis may amplify oxidative and inflammatory stress while promoting a tissue landscape more permissive to genomic instability and maladaptive repair [83,84,114].

### 8.4. Open Questions and Unresolved Mechanistic Links

In summary, the evidence presented in this review supports the hypothesis that endometriosis-associated dysbiosis may play a role in endometrial carcinogenesis via several overlapping mechanisms. These include the breakdown of the epithelial barrier, the activation of TLRs and NF-κB, the amplification of cytokines, macrophage and immune response dysfunction, the estrobolome-mediated changes in the recirculation of estrogen, and the ongoing oxidative and proliferative stress. Importantly, the proposed association is not one of straightforward direct causality but of biologic facilitation. Dysbiosis may set the stage for a permissive environment in which chronic inflammation, endocrine imbalance, molecular vulnerability, and immune control impairment converge to promote malignant transformation. From this perspective, endometriosis-associated dysbiosis should be regarded as a potentially important component of the broader pathophysiologic continuum linking chronic gynecologic inflammation to endometrial carcinogenesis [81,88,89,105,114,158,159,160,161]. The possible contribution of endometriosis-associated dysbiosis to endometrial carcinogenesis may not be uniform across molecular subtypes. The estrobolome-mediated increase in systemic estrogen exposure would, in principle, be expected to be most relevant to hormone-receptor-positive endometrioid tumours, particularly NSMP tumours in the ProMisE classification. Conversely, a permissive inflammatory and immune-evasive microenvironment could, in theory, facilitate the persistence of precursor lesions across several molecular subtypes, including p53-abnormal tumours in the ProMisE classification. To our knowledge, no prospective cohort study has stratified endometrial cancer microbiome data according to ProMisE molecular subtype. This remains an important gap that future contamination-aware and adequately powered studies should address.

## 9. Therapeutic and Translational Implications

At present, microbiome findings in endometriosis and endometrial cancer are more informative for biologic stratification than for immediate clinical intervention. Even so, the available evidence supports the translational value of incorporating microbial data into broader risk models that also include obesity, menopausal status, endocrine exposure, inflammatory markers, and molecular subtypes. In the near term, the most realistic application is likely to be biomarker development rather than microbiome-directed therapy as standard care [8,11,151].

Potential intervention strategies can nevertheless be outlined conceptually. These include diet-based modulation of the gut microbiota, restoration of SCFA-producing capacity, cautious exploration of probiotics or prebiotics, and future approaches aimed at modulating β-glucuronidase-related estrogen recirculation or dysbiosis-associated inflammatory signaling. For the reproductive tract, microbial profiling may eventually contribute to minimally invasive screening or to biologically informed surveillance in selected high-risk groups, although validation is still required [10,74,162,163,164]. Any therapeutic interpretation should remain cautious. The field lacks longitudinal trials, contamination-aware intervention studies, and validated microbial targets for endometrial cancer prevention or treatment. Microbiome-directed strategies should therefore be framed as emerging translational opportunities whose value will depend on rigorous functional validation, patient stratification, and integration with established endocrine, metabolic, and oncologic management [9,80,149]. Conceptually, microbiome-based approaches could complement existing gynaecological assessment. Endocervical or vaginal swabs, endometrial lavage fluid, or even first-void urine are technically feasible sampling routes and could be incorporated into routine annual gynaecological evaluation, provided that appropriate prospective studies first establish their predictive value. Specifically, before any clinical implementation, the following steps would be required: (1) standardisation of sampling, DNA extraction, and bioinformatics across centres; (2) validated contamination-aware pipelines; (3) establishment of prospective reference cohorts stratified by menopausal status, BMI, ethnicity, hormonal exposure, and histological/molecular subtype; (4) demonstration, in longitudinal cohorts, that a defined microbial signature predicts incident endometrial carcinoma with adequate sensitivity and specificity; and (5) demonstration of clinical utility, ideally against or alongside current screening strategies (e.g., transvaginal ultrasound and endometrial sampling in postmenopausal women with bleeding). For now, no such microbial test is clinically validated, and any proposal to incorporate microbiome-based screening into routine annual gynaecological visits must therefore be regarded as premature.

## 10. Limitations and Future Research

The present field remains methodologically fragile, and this limits the strength of biological inference. Much of the available evidence is derived from small and heterogeneous cohorts, cross-sectional study designs, and low-biomass sampling environments in which contamination can substantially affect microbial signal detection. Additional sources of variability include differences in sampling sites, timing of collection, DNA extraction methods, sequencing platforms, targeted 16S rRNA regions, bioinformatic pipelines, and analytical thresholds. These limitations make direct comparisons across studies difficult and reduce confidence in the reproducibility of reported microbial signatures. Accordingly, current evidence is more supportive of association and biologic plausibility than of direct causality.

Another important limitation is that taxonomic profiling alone does not establish functional relevance. In many studies, the inferred links between dysbiosis, inflammatory signaling, endocrine disruption, immune remodeling, and malignant progression remain indirect. Furthermore, the distinction between local reproductive tract dysbiosis and systemic gut-mediated effects is not always clear, and reverse causation cannot be excluded, particularly in established disease states. For these reasons, microbiome alterations should currently be interpreted as context-dependent correlates or potential modulators rather than as validated drivers of malignant transformation [6,9,10,11].

Future research should prioritize standardized and contamination-aware sampling strategies, prospective longitudinal cohorts, and stronger integration of microbiome profiling with metabolomics, transcriptomics, host immune characterization, and clinical phenotyping. Functional studies will be essential to determine whether specific microbial communities, metabolites, or host–microbe interactions contribute meaningfully to endometrial carcinogenesis or instead reflect secondary changes in the disease microenvironment. In translational terms, the most promising next steps include the development of reproducible biomarker panels, biologically informed risk stratification models, and mechanistically grounded intervention hypotheses that can be tested in well-designed human studies [7,8,73,77,161,162,163,164].

## 11. Conclusions

Current evidence does not establish a causal role of endometriosis-associated dysbiosis in uterine endometrial carcinogenesis. Rather, available data suggest a biologically plausible, context-dependent modulatory role that requires formal prospective investigation and is conceptually distinct from the better-established endometriosis-to-ovarian-carcinoma continuum. The microbiome is best interpreted as a context-dependent biologic modulator rather than a singular causal agent, with plausible effects on endocrine, inflammatory, immune, and metabolic pathways relevant to malignant transformation.

Although the translational potential of this field is considerable, present evidence is not sufficient to support direct microbiome-targeted clinical strategies as standard care. Future progress will depend on rigorous, contamination-aware longitudinal studies, on functional validation of specific microbial signals, and on integrating microbiome data with established endocrine, metabolic, immunologic, and oxidative biomarkers. The clinical translation of microbiome-based screening or risk stratification tools remains premature and requires well-designed prospective cohorts before any consideration of incorporation into routine gynaecological practice.

## Figures and Tables

**Table 1:** Studies evaluating microbiota alterations in endometriosis.

Author, Year	Disease Definition	Sample Size	Sample Type	Menopausal Status	BMI/Metabolic Profile	Sampling Collection Route	Antibiotic/Hormonal Exposure	Sequencing/Analytical Method	Contamination Controls	Main Findings	Key Limitations
Khan et al., 2018 [82]	Endometriosis vs. controls	Endometriosis = 73; controls = 69	Peritoneal fluid and menstrual blood samples	Predominantly reproductive-age; exact status NR	Not reported (NR)	Surgical peritoneal fluid collection; menstrual blood sampling	NR	Bacterial culture analysis; endotoxin measurement	NR	Women with endometriosis showed higher endotoxin concentrations and increased bacterial contamination, suggestive of bacterial contamination in this cohortin endometriosis.	Cross-sectional; culture-based; possible sampling contamination; no adjustment for BMI, hormones, or antibiotics. No reported adjustment for major confounders (e.g., age/menopausal status, BMI/metabolic factors, antibiotic or hormonal exposure, diet, and smoking).
Ata et al., 2019 [90]	Stage III/IV endometriosis vs. controls	Endometriosis stage III/IV = 14; controls = 14	Vaginal, cervical, and fecal samples	Predominantly reproductive-age, exact status NR	NR	Vaginal/cervical swabs and stool collection	NR	16S rRNA sequencing	NR	This study reported reduced Lactobacillus dominance and increased microbial diversity across gut and lower reproductive tract samples in women with endometriosis; however, findings were compartment-specific and should not be interpreted as a uniform disease signature.	Mixed anatomical compartments; limited confounder control. Residual confounding by BMI, hormonal exposure, diet, and smoking cannot be excluded.
Hernandes et al., 2020 [97]	Endometriosis vs. controls	Endometriosis = 66; controls = 198	Fecal microbiota samples	Predominantly reproductive-age; exact status NR	NR	Stool collection	NR	Microbiome profiling and diversity analysis	Not applicable for low-biomass contamination; other controls NR	This study reported altered gut microbial composition in endometriosis, including differences in Firmicutes and Bacteroidetes abundance, but causal interpretation is limited by the observational design.	Stool-only study; no reproductive tract sampling; cross-sectional; diet/BMI confounding not clearly addressed. Partial adjustment for confounding; residual confounding by age, BMI/metabolic profile, hormonal exposure, diet, or smoking cannot be excluded.
Svensson et al., 2021 [86]	Endometriosis vs. controls	Endometriosis = 25; controls = 25	Endometrial and vaginal swabs	Predominantly reproductive-age; exact status NR	NR	Vaginal and endometrial sampling; exact route NR	NR	16S rRNA sequencing and diversity analysis	NR	This study described differences in vaginal and endometrial microbial composition between endometriosis cases and controls, although the precise taxonomic alterations require cautious interpretation because of low-biomass sampling and anatomical heterogeneity.	mixed compartments; low-biomass contamination risk; limited confounder adjustment. Limited adjustment for major confounders.
Shan et al., 2021 [72]	Endometriosis vs. controls	Endometriosis = 31; controls = 15	Fecal samples	Predominantly reproductive-age; exact status NR	NR	Stool collection	NR	16S rRNA sequencing and taxonomic profiling	Not applicable for low-biomass contamination; other controls NR	Endometriosis was shown to have a modified Firmicutes/Bacteroidetes ratio and an increase in inflammatory-associated taxa.	Stool-only analysis; cross-sectional design. Findings may be influenced by diet, BMI/metabolic factors, antibiotic exposure, and smoking.
Wei et al., 2020 [98]	Endometriosis vs. controls	Endometriosis = 36; controls = 14	Endometrial and vaginal samples	Predominantly reproductive-age; exact status NR	NR	Endometrial and vaginal sampling; exact route NR	NR	Microbial sequencing combined with inflammatory marker assessment	NR	Microbiome alterations correlated with inflammatory cytokine expression and disease severity.	Mixed sample types; low-biomass concerns; no longitudinal follow-up. Residual confounding by hormonal treatment, menstrual phase, BMI, diet, and smoking cannot be excluded.
Wessels et al., 2021 [91]	Endometriosis vs. controls	Endometriosis = 21; controls = 19	Endometrial biopsies	Predominantly reproductive-age; exact status NR	NR	Endometrial biopsy	NR	16S rRNA sequencing	NR	Reported increased endometrial microbial diversity in this study and reduced Lactobacillus abundance.	Low-biomass contamination risk; contamination-aware workflow not clearly reported. Limited reporting of confounder control.
Chang et al., 2022 [99]	Endometriosis vs. controls	Endometriosis = 54; controls = 34	Cervical swab samples	Predominantly reproductive-age; exact status NR	NR	Cervical swab sampling	NR	16S rRNA sequencing	NR	Specific cervical microbial profiles were reported to correlate with disease severity and infertility in this endometriosis cohort, but external validation is lacking.	Cervical compartment only; cross-sectional; limited relevance to uterine microbiota. Microbiome differences may reflect local compartment effects and unmeasured confounders including hormonal status, diet, and smoking.
MacSharry et al., 2024 [100]	Endometriosis vs. controls	Endometriosis = 44; controls = 38	Vaginal and urine samples	Predominantly reproductive-age; exact status NR	NR	Vaginal swab and urine collection	NR	Microbiome and virome sequencing	NR	This study reported depletion of Lactobacillus species and enrichment of Streptococcus and Proteobacteria in vaginal and urine samples from women with endometriosis; these observations remain exploratory and require replication.	Mixed biospecimens; exploratory design; unclear relevance of urine; confounder control unclear. Residual confounding by antibiotic exposure, sexual activity, diet, and smoking remains possible.
Qing et al., 2024 [101]	Meta-analysis of endometriosis-associated vaginal microbiome studies	Meta-analysis including multiple studies	Vaginal microbiome datasets	Mixed/NR across included cohorts	Mixed/NR across included cohorts	Derived from published studies	Mixed/NR across included studies	Systematic review and meta-analysis	Dependent on controls in included studies	The meta-analysis suggested an overall association between endometriosis and vaginal dysbiosis, including reduced Lactobacillus and increased inflammatory-associated microorganisms, but study heterogeneity remained substantial.	Marked inter-study heterogeneity; variable pipelines and populations; limited causal inference. Adjustment for confounding was not clearly reported.

**Table 2:** Studies evaluating microbiota alterations in endometrial cancer.

Author and Year	Disease Definition	Sample Size	Sample Type	Menopausal Status	BMI/Metabolic Profile	Sample Collection Route	Antibiotic/Hormonal Exposure	Sequencing/Analytical Method	Contamination Controls	Main Findings	Key Limitations
Walther-António et al., 2016 [10]	Endometrial cancer (EC) and endometrial hyperplasia (EH) vs. benign conditions	EC = 17; EH = 4; benign = 10	Vaginal, cervical, uterine, fallopian tube, and ovarian specimens	Mixed/NR	NR	Multi-site reproductive tract sampling during surgery	NR	16S rDNA amplicon sequencing (V3–V5 region, Illumina MiSeq)	NR	In this cohort, EC and EH were reported to differ from benign conditions in reproductive tract microbial composition. Co-detection of Atopobium vaginae and a Porphyromonas species (closely related to P. somerae), together with elevated vaginal pH, was associated with EC, but these findings should not be interpreted as a validated diagnostic signature.	Mixed compartments; inclusion of EH may blur interpretation; contamination controls not clearly described. Limited control for menopause, BMI/metabolic status, and contamination risk.
Walsh et al., 2019 [11]	Hysterectomy cohort including EC cases	Total cohort = 151 women undergoing hysterectomy	Vaginal, cervical, uterine, tubal, and ovarian samples	Mixed; postmenopausal status identified as major driver	NR	Multi-site reproductive tract sampling	NR	16S rRNA gene sequencing (V3–V5 region); qPCR validation for Porphyromonas somerae; network-based analysis	NR	This study identified an EC-associated polymicrobial network (“ECbiome”), although the observed pattern appeared to be strongly influenced by postmenopausal status. Porphyromonas somerae emerged as a leading cohort-specific predictor, but the finding has not been established as a reproducible biomarker across independent studies.	Menopause strongly confounds findings; mixed compartments; observational design. Partial adjustment for confounding; residual confounding by age, BMI/metabolic profile, hormonal exposure, diet, or smoking cannot be excluded.
Gressel et al., 2021 [141]	Endometrioid EC and uterine serous carcinoma vs. controls	Controls = 10; endometrioid EC = 14; uterine serous carcinoma = 11	Endometrial, cervicovaginal, and anorectal swabs	NR	NR	Intraoperative swab sampling	NR	16S rRNA gene sequencing (V4); QIIME2; ANCOM; PICRUSt	NR	This study reported niche-specific microbial differences across endometrial, cervicovaginal, and anorectal compartments, with uterine serous carcinoma showing a distinct profile. The results support the possibility of subtype-specific variation but remain limited by sample size.	Mixed compartments; predicted rather than directly measured function. Limited adjustment for BMI, menopausal status, and other clinical confounders.
Li et al., 2021 [142]	EC vs. controls; paired tumor and adjacent non-tumor tissue	EC = 30; controls = 10	Endometrial tissue; paired tumor and adjacent non-tumor tissue for transcriptomics	NR	NR	Prospective endometrial tissue sampling	NR	16S rRNA sequencing; paired RNA-seq; correlation with hematologic biomarkers	NR	Prevotella and Pelomonas were reported to be more prevalent in EC tissue in this study, and Prevotella abundance correlated with coagulation-related host biomarkers; however, the observational design precludes causal inference.	Low-biomass tissue setting; limited confounder adjustment. Adjustment for confounding was not clearly reported.
Chen et al., 2021 [143]	EC vs. controls	EC = 9; controls = 8	Endometrial biopsies	NR	NR	Endometrial biopsy sampling	NR	Metatranscriptomic sequencing; host transcriptomic profiling; HUMAnN3; GSEA; O2PLS integration	NR	This metatranscriptomic study identified functionally active microbial species associated with EC and linked them to host pathways involved in migration and signaling. These findings support biologic plausibility but require validation in larger contamination-aware cohorts.	Very small sample size; low-biomass setting; exploratory design; no external validation. No reported adjustment for major confounders (e.g., age/menopausal status, BMI/metabolic factors, antibiotic or hormonal exposure, diet, and smoking).
Lu et al., 2021 [70]	EC vs. benign uterine lesions	EC = 25; benign uterine lesions = 25	Endometrial tissues	NR	NR	Endometrial tissue sampling	NR	16S rRNA gene sequencing; qPCR and Western blot for IL-6, IL-8, and IL-17	NR	EC samples in this cohort showed higher microbial diversity and relative enrichment of Micrococcus, which correlated with inflammatory cytokine expression. The results suggest a microbiota–inflammation association but do not establish causality.	Heterogeneous benign comparator; low-biomass contamination risk. Residual confounding by menopause, obesity, and treatment exposure is possible.
Burkett et al., 2022 [144]	Early-stage EC cohort	Early-stage EC = 95 (Black women = 23; White women = 72)	Banked tumor specimens	NR	Obesity-related differences evaluated	Banked tumor tissue	NR	Tumor tissue microbiota profiling by bacterial 16S rRNA sequencing; comparison with TCGA-derived microbial data	NR	This study reported racial and obesity-related differences in tumor-associated microbial profiles, suggesting that host factors may substantially influence observed EC microbiome patterns.	No benign control group in summary; banked tissue pre-analytic variability; causality not inferable. Potential confounding by race, BMI, treatment history, and batch effects.
Wang et al., 2022 [130]	EC vs. adjacent pericancer endometrium	EC = 28 postmenopausal women	Paired EC and adjacent pericancer endometrial tissues	Postmenopausal only	Vaginal pH and Lactobacillus associations reported; broader metabolic profile NR	Paired hysterectomy tissue sampling	NR	16S rRNA sequencing	NR	Paired EC and pericancer tissues showed enrichment of several anaerobic genera, including Prevotella, Atopobium, Anaerococcus, Dialister, Porphyromonas, and Peptoniphilus. These findings support within-cohort ecological differences but are not sufficient to define a reproducible EC signature.	Postmenopausal-only cohort; no external benign controls; low-biomass tissue sampling. Partial adjustment for confounding; residual confounding by age, BMI/metabolic profile, hormonal exposure, diet, or smoking cannot be excluded.
Chao et al., 2022 [145]	EC/EH vs. controls	Discovery cohort: 35 lavage specimens from 32 women; validation cohort: EC/EH = 46, controls = 13	Endometrial lavage fluid	NR	NR	Office hysteroscopy-guided lavage	NR	16S rRNA sequencing (V3–V4); qPCR validation; computational functional prediction	NR	This study reported higher microbial diversity and reduced Lactobacillus/Bifidobacterium abundance in EC/EH lavage samples, together with predicted metabolic pathway differences. Findings remain exploratory.	Mixed EC and EH groups; lavage-specific methodology; predicted rather than measured function. Partial adjustment for confounding; residual confounding by age, BMI/metabolic profile, hormonal exposure, diet, or smoking cannot be excluded.
Barczyński et al., 2023 [146]	EC, atypical hyperplasia, and benign disease	EC = 48; atypical hyperplasia = 21; benign disease = 27	Paired vaginal and cervical swabs	NR	NR	Prospective vaginal and cervical swab sampling	NR	molecular detection/qPCR-based profiling of 19 microorganisms	NR	Cancer-associated cervical/vaginal clusters were characterized in this study by lower lactobacilli and enrichment of potentially pro-inflammatory taxa; however, interpretation is limited by targeted profiling and lack of full metagenomic resolution.	Targeted organism panel only; no endometrial samples; lower tract findings may not reflect uterine compartment. Residual confounding by vaginal ecology, hormones, diet, and smoking is possible.
Leoni et al., 2024 [147]	EC vs. benign polymyomatous uterus	EC = 8; benign polymyomatous uterus = 6	Endometrial tissue biopsies from two intrauterine sites	NR	NR	Sterile hysterectomy-derived biopsies	NR	droplet digital PCR for bacterial load; NGS metabarcoding	Bacterial load quantification and sterile sampling reported	This study confirmed that the endometrium is a low-biomass niche with marked site-specific variability and reported several genera exclusively in EC samples. These findings underscore the need for strict contamination-aware interpretation.	Very small sample size; marked site-specific variability; no external validation. No reported adjustment for major confounders (e.g., age/menopausal status, BMI/metabolic factors, antibiotic or hormonal exposure, diet, and smoking).
Han et al., 2024 [148]	EC, EH, and benign controls	EC = 33; EH = 15; benign controls = 15	Endometrial tissue	NR	Estrogen-related metabolite correlations reported; BMI/metabolic profile NR	Prospective tissue sampling	NR	16S rRNA sequencing (V3–V4), ITS1 fungal sequencing, electron microscopy, and LC-MS metabolomics	NR	This study reported increased bacterial and fungal diversity in EC/EH, including Penicillium enrichment and correlations with inflammatory and estrogen-related metabolites. These multi-omic associations are biologically interesting but remain cohort-specific.	Mixed EC/EH cohort; moderate sample size; low-biomass sampling issues. Adjustment for confounding was not clearly reported.
Semertzidou et al., 2024 [149]	EC vs. benign controls	EC = 37; benign controls = 24	Vagina, cervix, endometrium, fallopian tubes, ovaries, and rectum	NR	NR	Contamination-controlled multisite sampling	NR	16S rRNA sequencing (V1–V2); 16S qPCR; organoid assays with Lactobacillus crispatus conditioned media	Contamination-controlled multisite sampling reported	Reduced Lactobacillus abundance, increased diversity, and enrichment of anaerobic taxa were associated with EC across multiple anatomical sites in this contamination-controlled study. Organoid experiments suggested anti-proliferative effects of Lactobacillus crispatus conditioned medium, but translational significance remains preliminary.	Cross-sectional; mixed compartments; organoid findings are not equivalent to clinical proof. Partial adjustment for confounding; residual confounding by age, BMI/metabolic profile, hormonal exposure, diet, or smoking cannot be excluded.
Jimenez et al., 2025 [150]	Grade 1/2 endometrioid EC, other EC, EH, and benign controls	Total cohort = 192 (grade 1/2 endometrioid EC = 53; other EC = 13; EH = 18; benign = 108)	Vaginal and rectal swabs	NR	NR	Vaginal and rectal swab sampling	NR	16S rRNA amplicon sequencing; QIIME2; ANCOM-BC; PICRUSt2; microbial network analysis	NR	This study reported depletion of protective vaginal and rectal taxa and enrichment of anaerobic genera in EC, together with altered predicted metabolic pathways. The findings suggest mucosal cross-talk but do not establish a uniform disease signature.	No endometrial tissue sampled; lower tract/rectal compartments only; predicted function. Partial adjustment for confounding; residual confounding by age, BMI/metabolic profile, hormonal exposure, diet, or smoking cannot be excluded.
Kuźmycz et al., 2025 [151]	EC vs. myoma controls	EC = 16; myoma controls = 13	Endocervical canal swabs; functional cell assays	NR	NR	Endocervical swab sampling	NR	16S rRNA sequencing (V3–V4); QIIME2; in vitro adhesion and ROS assays in human uterine fibroblasts	NR	Endocervical samples from EC cases showed higher diversity and enrichment of anaerobic genera, and in vitro assays demonstrated adhesion and ROS generation by Anaerococcus vaginalis. These data support biologic plausibility but remain preliminary and experimental.	Endocervical rather than endometrial compartment; experimental findings not equal to in vivo proof. Residual confounding remains possible.

## Data Availability

No new data were created or analyzed in this study. Data sharing is not applicable to this article.

## Abbreviations

The following abbreviations are used in this manuscript:

Akt	Protein kinase B
ARID1A	AT-rich interaction domain-containing protein 1A
EMT	Epithelial–mesenchymal transition
FXR	Farnesoid X receptor
IL	Interleukin
JAK	Janus kinase
KRAS	Kirsten rat sarcoma viral oncogene homolog
LPS	Lipopolysaccharide
MAPK	Mitogen-activated protein kinase
MMPs	Matrix metalloproteinases
MMRd	Mismatch repair-deficient
MSI-H	Microsatellite instability-high
NF-κB	Nuclear Factor kappa B
NLRs	NOD-like receptors
NSMP	No specific molecular profile
mTOR	Mechanistic target of rapamycin
PI3K	Phosphoinositide 3-kinase
PTEN	Phosphatase and tensin homolog
RAS	Rat sarcoma viral oncogene
ROS	Reactive oxygen species
SCFAs	Short-chain fatty acids
STAT	Signal Transducer and Activator of Transcription
TGR5	Takeda G protein-coupled receptor 5
TLRs	Toll-like receptors
TNF-α	Tumor necrosis factor alpha
uNK	Uterine natural killer
